# Supplementary material for: Pan-neutralization of parainfluenza viruses by a hemagglutinin-neuraminidase antibody
Source: bioRxiv. 2026 Sep 10:2026.09.07.749649. Preprint. [Version 1] doi: 10.64898/2026.09.07.749649 (PMC13622509; doi:10.64898/2026.09.07.749649)
Supplement: Supplementary file 1 [file NIHPP2026.09.07.749649V1-supplement-1.pdf]

bioRxiv preprint doi: https://doi.org/10.64898/2026.09.07.749649; this version posted September 10, 2026. The copyright holder for this preprint (which was not certified by peer review) is the author/funder, who has granted bioRxiv a license to display the preprint in perpetuity. It is made available under a CC-BY 4.0 International license.

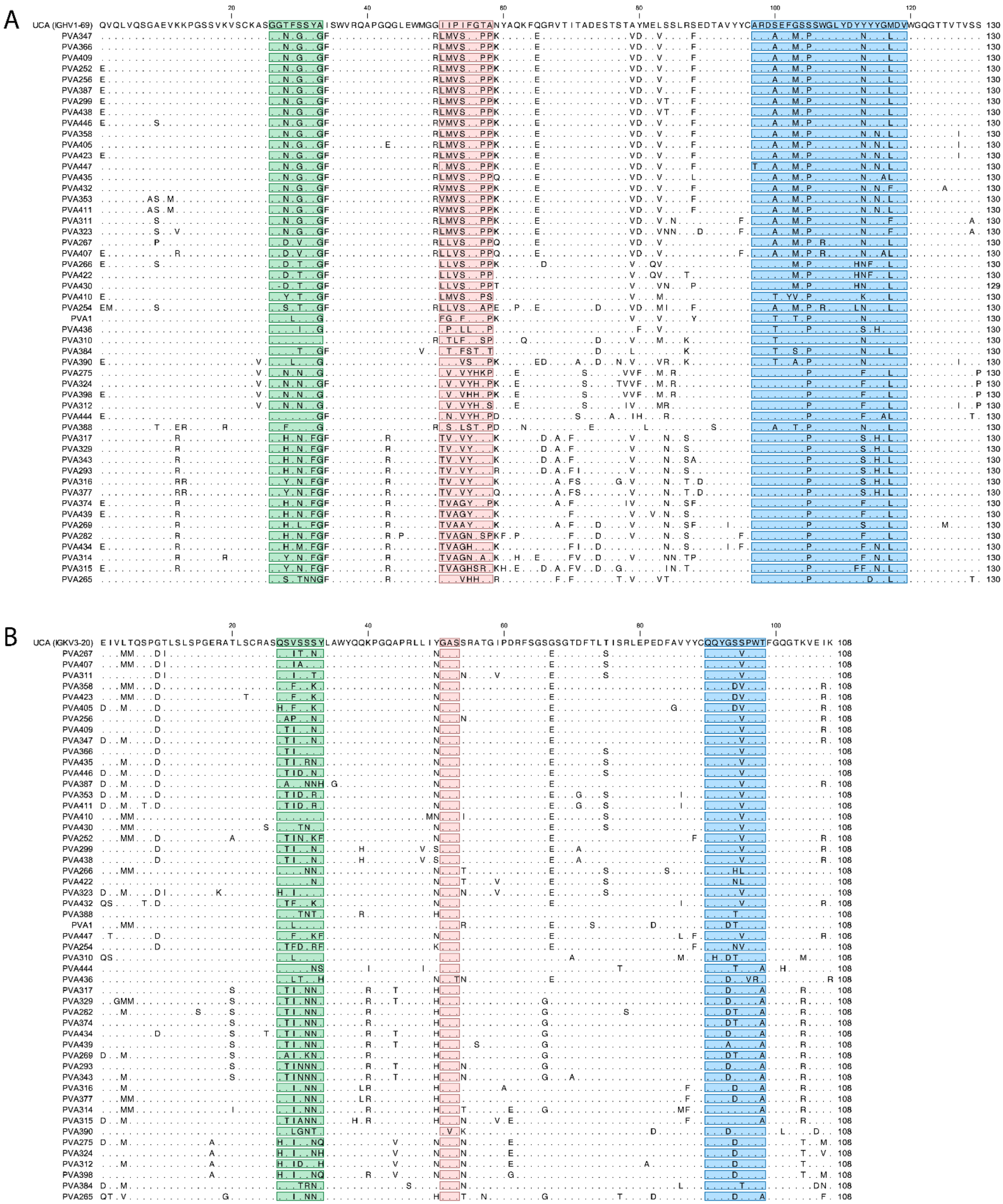

Figure S1. Amino acid sequence alignment of PVA269 clonal family members. Sequence alignment of the VH (A) and VL (B) genes of the 51 mAbs representing the PVA clonal family with respective unmutated common ancestors (UCAs). Complementary determining regions (CDRs) for the heavy and light chains are highlighted in green

bioRxiv preprint doi: https://doi.org/10.64898/2026.09.07.749649; this version posted September 10, 2026. The copyright holder for this preprint (which was not certified by peer review) is the author/funder, who has granted bioRxiv a license to display the preprint in perpetuity. It is made available under a CC-BY 4.0 International license.

(CDR1), red (CDR2), and blue (CDR3). Residues that are identical to UCAs are shown as dots.

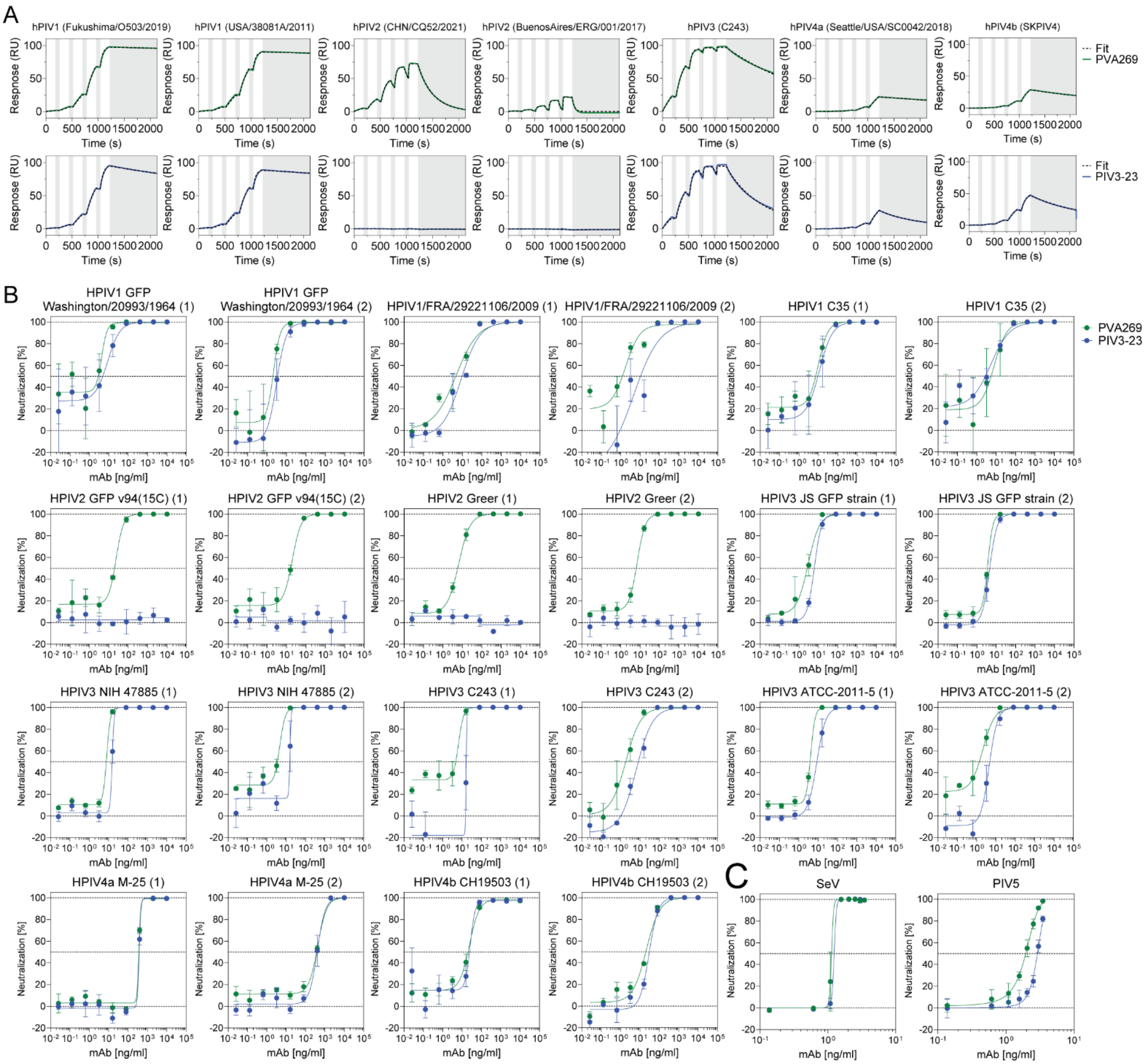

Figure S2. Evaluation of the PVA269 and PIV3-23 cross-reactivity and neutralization breadth. A. SPR sensorgrams of PVA269 or PIV3-23 recombinant Fabs binding to HPIV1-4 HN glycoproteins immobilized at the surface of SPR chips. Gray blocks denote the dissociation phases during single cycle kinetics experiments. **B,C**. Dose-response curves for the analysis of PVA269- and PIV3-23-mediated neutralization of a panel of HPIV 1-4 clinical isolates (B) and animal viruses (C) using LLC-MK2 target cells. n=2 independent biological experiments are reported for each clinical strain. Each independent experiment was performed with at least 3 technical replicates.

bioRxiv preprint doi: https://doi.org/10.64898/2026.09.07.749649; this version posted September 10, 2026. The copyright holder for this preprint (which was not certified by peer review) is the author/funder, who has granted bioRxiv a license to display the preprint in perpetuity. It is made available under a CC-BY 4.0 International license.

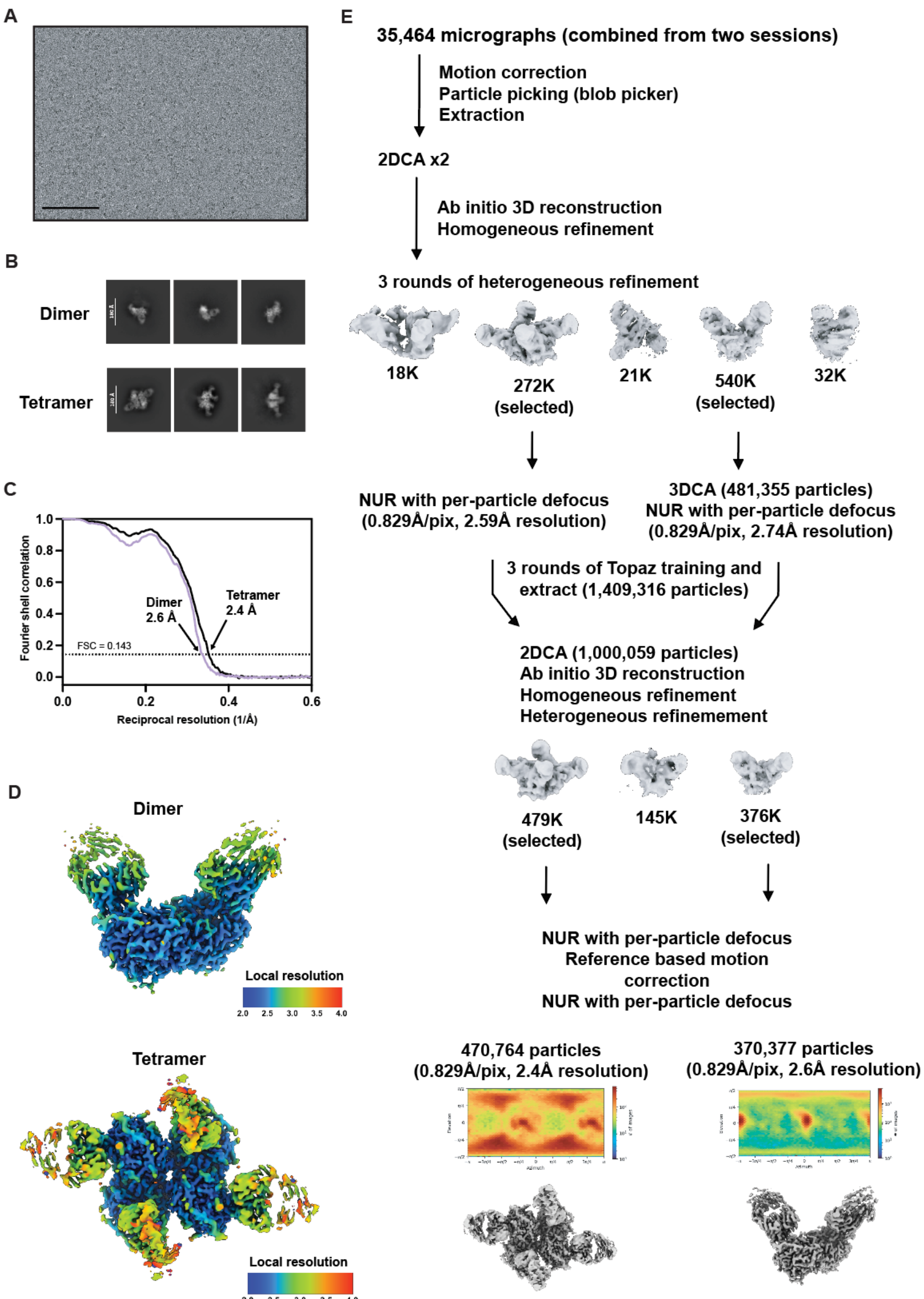

Figure S3. CryoEM data collection and processing of the PVA269-bound HPIV2 HN dataset. A-B. Representative electron micrograph and 2D class averages of the

bioRxiv preprint doi: https://doi.org/10.64898/2026.09.07.749649; this version posted September 10, 2026. The copyright holder for this preprint (which was not certified by peer review) is the author/funder, who has granted bioRxiv a license to display the preprint in perpetuity. It is made available under a CC-BY 4.0 International license.

PVA269-bound HPIV2 HN dimer and tetramers embedded in vitreous ice. Scale bars: 100 nm (A) and 180 Å (B). (C) Gold-standard Fourier shell correlation curve of PVA269-bound HPIV2 dimer and tetramer structures. The 0.143 cutoff is indicated by a horizontal dashed line. (D) Local resolution estimation of the PVA269-bound HPIV2 dimer and tetramer reconstructions calculated using cryoSPARC[26] and plotted on the corresponding sharpened maps. (E) Data processing flowchart. NUR: non-uniform refinement. The angular distribution of particle images calculated using cryoSPARC is shown as a heat map for each reconstruction.

bioRxiv preprint doi: https://doi.org/10.64898/2026.09.07.749649; this version posted September 10, 2026. The copyright holder for this preprint (which was not certified by peer review) is the author/funder, who has granted bioRxiv a license to display the preprint in perpetuity. It is made available under a CC-BY 4.0 International license.

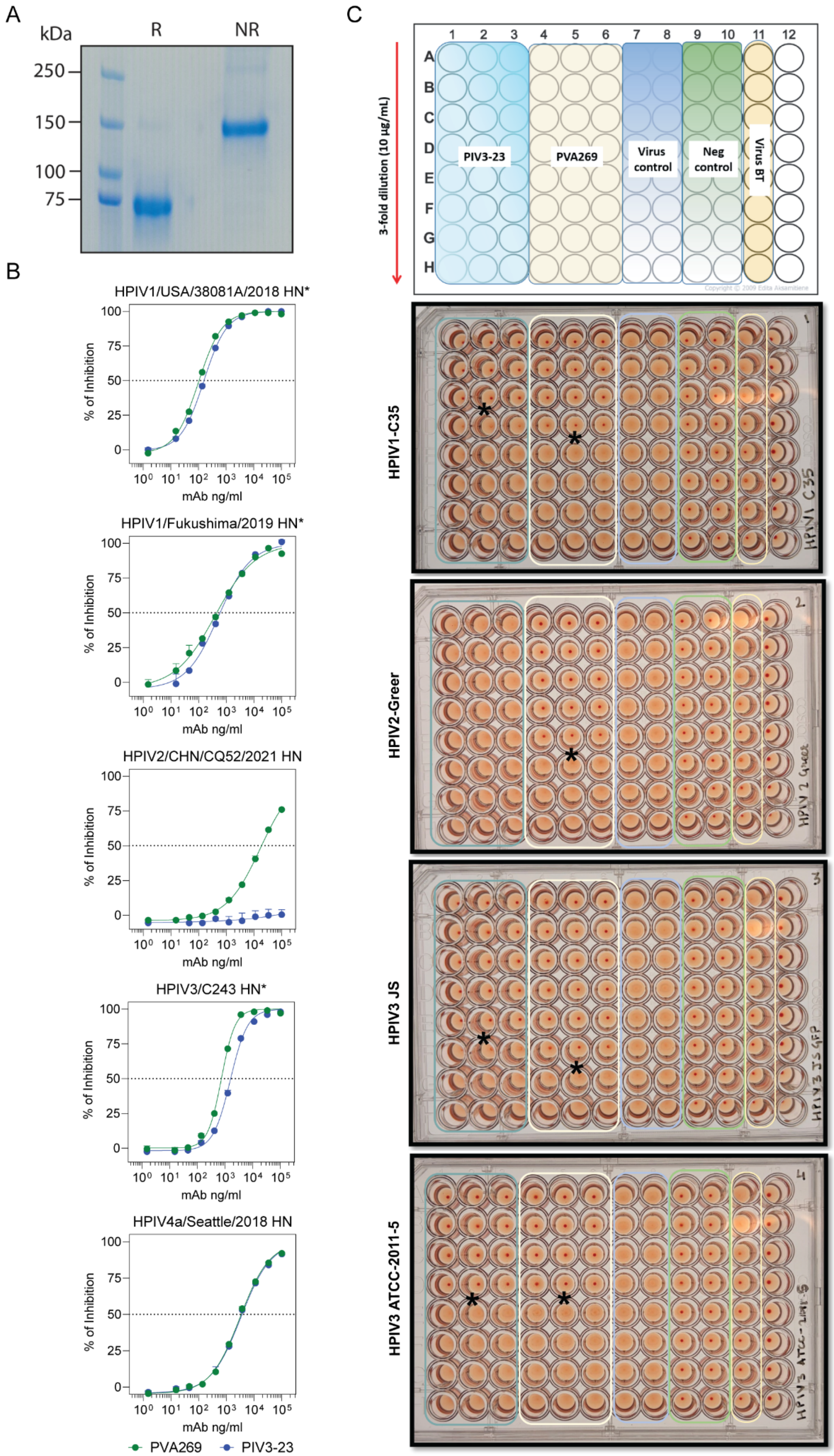

bioRxiv preprint doi: https://doi.org/10.64898/2026.09.07.749649; this version posted September 10, 2026. The copyright holder for this preprint (which was not certified by peer review) is the author/funder, who has granted bioRxiv a license to display the preprint in perpetuity. It is made available under a CC-BY 4.0 International license.

Fig S4. Evaluation of HN oligomerization and mAb-mediated HN inhibition. A. Reducing and non-reducing SDS-PAGE of the purified recombinant HPIV2 HN ectodomain used for structural studies. **B**. PVA269- and PIV3-23-mediated inhibition of HPIV1-4 HN (*head or full ectodomain) sialidase activity as measured using a MUNANA assay. **C**. PVA269- and PIV3-23-mediated inhibition of HPIV1-3 hemagglutination of guinea pig red blood cells. Asterisks mark the greatest mAb dilution at which HAI activity was observed.

bioRxiv preprint doi: https://doi.org/10.64898/2026.09.07.749649; this version posted September 10, 2026. The copyright holder for this preprint (which was not certified by peer review) is the author/funder, who has granted bioRxiv a license to display the preprint in perpetuity. It is made available under a CC-BY 4.0 International license.

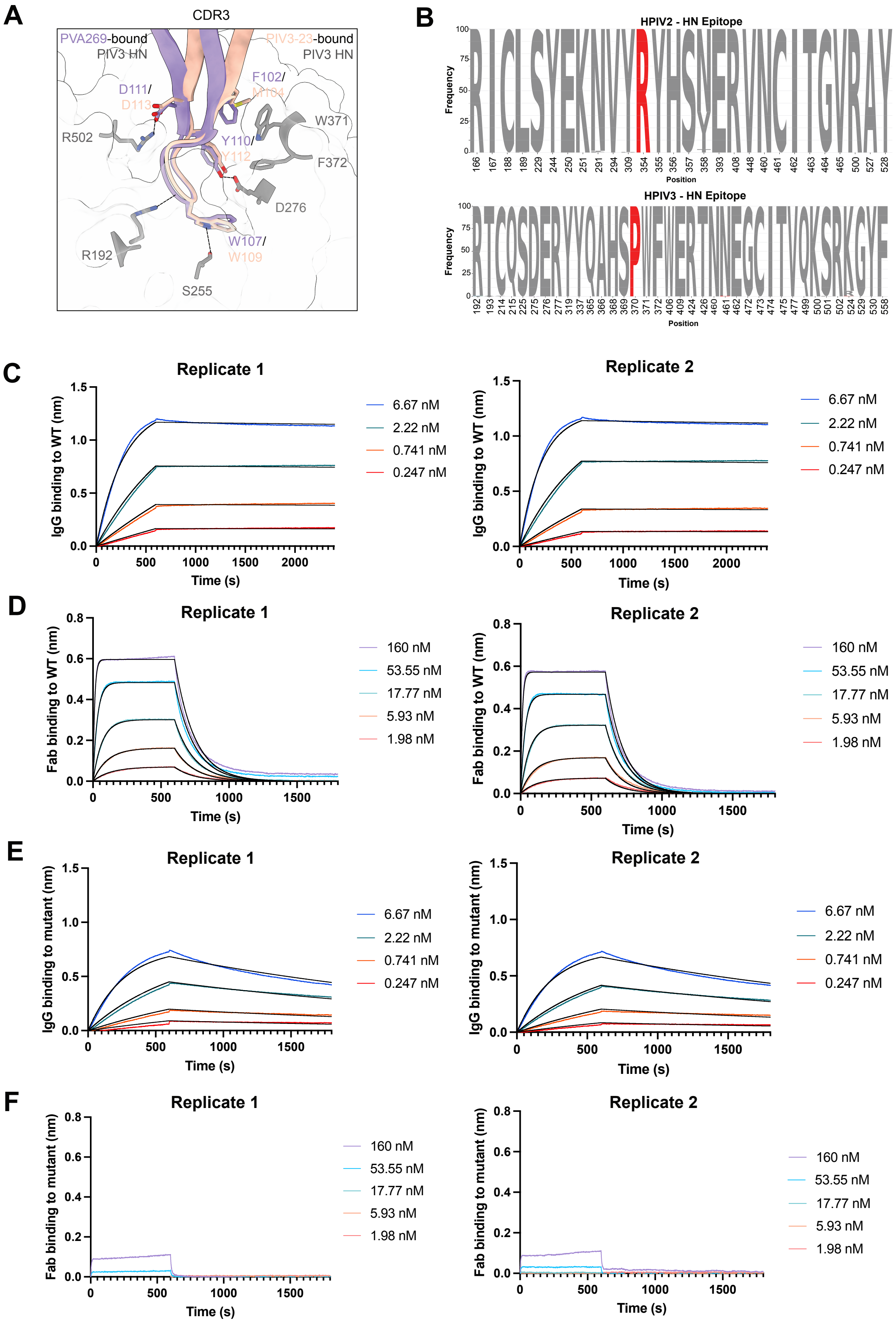

bioRxiv preprint doi: https://doi.org/10.64898/2026.09.07.749649; this version posted September 10, 2026. The copyright holder for this preprint (which was not certified by peer review) is the author/funder, who has granted bioRxiv a license to display the preprint in perpetuity. It is made available under a CC-BY 4.0 International license.

Fig S5. Comparison of PVA269 and PIV3-23 binding to HN, epitope conservation and evaluation of PVA269 binding to wildtype and mutant HPIV2 HN. A. Zoomed-in view of PVA269 (purple) and PIV3-23 (orange, PDB: 8TQI) CDRH3 from their respective HPIV3-bound structures. Interacting residues are labeled in purple for PVA269, orange for PIV3-23, and dark grey for HPIV3. **B** Logo plot amino acid conservation of PVA269 epitope based on publicly available HN sequences from HPIV2 (n = 210) and HPIV3 (n = 2140). HPIV2 residue R354 and HPIV3 residue P370 are indicated in red. **C-F**. BLI binding analysis of PVA269 IgG (C) or Fab (D) to immobilized wildtype HPIV2 HN or of PVA269 IgG (E) or Fab (F) to immobilized R354A HPIV2 HN. Two biological replicates carried out with independently produced batches of HPIV2 HN are shown. The sensorgrams are colored according to the color key and the fit to the data using a 1:1 global fitting model is shown in black.

bioRxiv preprint doi: https://doi.org/10.64898/2026.09.07.749649; this version posted September 10, 2026. The copyright holder for this preprint (which was not certified by peer review) is the author/funder, who has granted bioRxiv a license to display the preprint in perpetuity. It is made available under a CC-BY 4.0 International license.

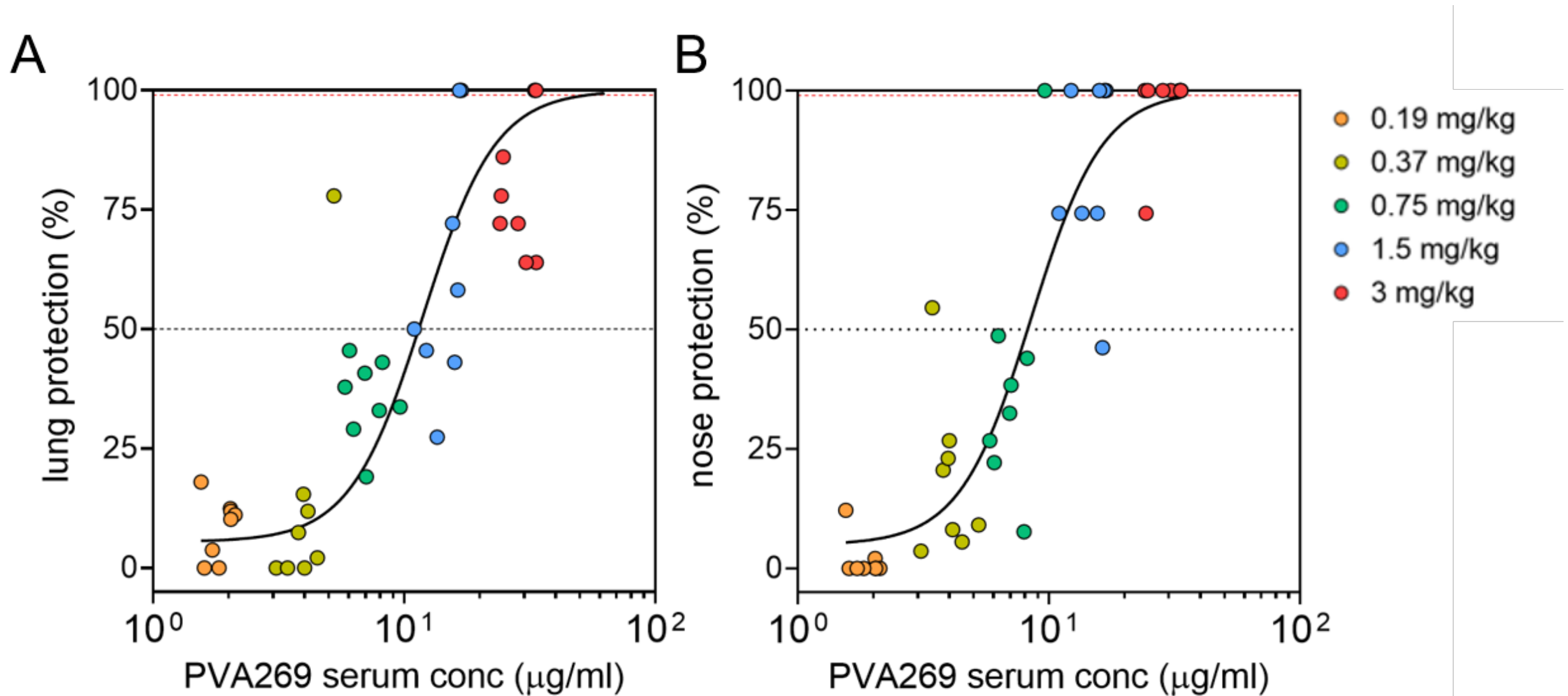

Fig S6. Correlation between PVA269 serum concentrations and protection of cotton rats against HPIV3 challenge. A-B, Non-linear regression fitting of lung (A) and nose (B) protection (derived from replicating viral titers) in function of PVA269 serum levels for the animal groups used in the challenge study. EC_{50} and EC_{99} are shown as black and red dotted lines, respectively. Replicating viral titers (protection) observed for the isotype-control antibody and the limit of detection of the plaque assays were used as 0% and 100% reference for data normalization, respectively.

bioRxiv preprint doi: https://doi.org/10.64898/2026.09.07.749649; this version posted September 10, 2026. The copyright holder for this preprint (which was not certified by peer review) is the author/funder, who has granted bioRxiv a license to display the preprint in perpetuity. It is made available under a CC-BY 4.0 International license.

Analyte	Ligand	K_D (nM)	k_{on} (1/Ms)	k_{off} (1/s)
PVA269-rFab	HPIV1-HN-Fukushima/O503/2019	0.25	6.67E+04	1.69E-05
	HPIV1-HN-USA/38081A/2011	0.24	6.98E+04	1.69E-05
	HPIV2-HN-BuenosAires//001/2017	35.7	6.75E+05	2.41E-02
	HPIV2-HN-CHN/CQ52/2021	7.9	4.63E+05	3.66E-03
	HPIV3-HN-C243-head	0.45	1.33E+06	5.95E-04
	HPIV4a-HN-Seattle/USA/SC0042/2018	25.4	1.12E+04	2.84E-04
	HPIV4b-HN-SKPIV4	19.1	2.20E+04	4.21E-04
PIV3-23-rFab	HPIV1-HN-Fukushima/O503/2019	2.4	5.74E+04	1.38E-04
	HPIV1-HN-USA/38081A/2011	0.82	6.69E+04	5.46E-05
	HPIV2-HN-BuenosAires//001/2017	NB	-	-
	HPIV2-HN-CHN/CQ52/2021	NB	-	-
	HPIV3-HN-C243	1.2	1.25E+06	1.53E-03
	HPIV4a-HN-Seattle/USA/SC0042/2018	69.1	3.46E+04	2.39E-03
	HPIV4b-HN-SKPIV4	17.7	4.23E+04	7.47E-04

NB – no binding observed

Table S1. Kinetics parameters of PVA269 or PIV3-23 recombinant Fabs to different HPIV-HN antigens from SPR binding assays.

bioRxiv preprint doi: https://doi.org/10.64898/2026.09.07.749649; this version posted September 10, 2026. The copyright holder for this preprint (which was not certified by peer review) is the author/funder, who has granted bioRxiv a license to display the preprint in perpetuity. It is made available under a CC-BY 4.0 International license.

	**PVA269-bound HPIV2 HN dimer<br>PDB 37JG<br>EMD 78229**	**PVA269-bound HPIV2 HN tetramer<br>PDB 37JH<br>EMD 78230**
**Data collection and processing**		
**Magnification**	105,000	
**Voltage (kV)**	300	
**Electron exposure (e–/Å^2)**	30.7	
**Defocus range (μm)**	-0.6 - -1.6	
**Pixel size (Å)**	0.829	
**Symmetry imposed**	C2	
**Initial particle images (no.)**	2,211,257	
**Final particle images (no.)**	370,377	470,764
**Map resolution (Å)**<br>**FSC threshold**	2.6<br>0.143	2.4<br>0.143
**Refinement**		
**Model resolution (Å)**<br>**FSC threshold**	2.8<br>0.5	2.7<br>0.5
**Map sharpening *B* factor (Å^2)**	-93.7	-82.9
**Model composition**<br>**Non-hydrogen atoms**<br>**Protein residues**<br>**Ligands**<br>**Water**	 11098<br>1364<br>46<br>292	 20374<br>2552<br>86<br>496
***B* factors (Å^2)**<br>**Protein**<br>**Ligand**<br>**Water**	 31.58<br>69.45<br>21.14	 102.38<br>152.66<br>88.62
**R.m.s. deviations**<br>**Bond lengths (Å)**<br>**Bond angles (°)**	 0.003<br>0.571	 0.004<br>0.628
**Validation**<br>**MolProbity score**<br>**Clashscore**<br>**Poor rotamers (%)**	 1.58<br>6.53<br>0.60	 1.46<br>5.36<br>0.70
**Ramachandran plot**<br>**Favored (%)**<br>**Allowed (%)**<br>**Disallowed (%)**	 96.59<br>3.41<br>0	 97.02<br>2.98<br>0

bioRxiv preprint doi: https://doi.org/10.64898/2026.09.07.749649; this version posted September 10, 2026. The copyright holder for this preprint (which was not certified by peer review) is the author/funder, who has granted bioRxiv a license to display the preprint in perpetuity. It is made available under a CC-BY 4.0 International license.

Table S2. CryoEM data collection, processing and model building statistics for the PVA269 Fab-bound HPIV2 HN structure.

	**PVA269-bound HPIV3 HN head**
**Data collection and processing**	
**Wavelength (Å)**	1
**Space group**	P 21 21 2
**Cell dimensions a, b, c (Å)**	84.42, 103.3, 115.2
**Cell angles α,β,γ (˚)**	90, 90, 90
**Resolution range (Å)**	43.22 - 1.9 (1.93-1.9)
**R-merge**<br>**R-meas**<br>**R-pim**	1.78e-17 (2.28e-17)<br>2.52e-17 (3.2e-17)<br>1.78e-17 (2.28e-17)
**CC1/2**<br>**CC***	1 (1)<br>1 (1)
**I/σ(I)**	2.85 (0.39)
**Completeness (%)**	85.03 (90.09)
**Multiplicity**	1.0 (1.0)
**Total reflections**	178579 (7638)
**Unique reflections**	172368 (7450)
**Wilson B-factor**	26.91
**Refinement**	
**Reflections used In refinement**<br>**For R-free**	 68005 (2845)<br>3466 (150)
**Rwork**<br>**Rfree**	0.23 (0.303)<br>0.28 (0.363)
***B* factors (Å²)**<br>**Protein**<br>**Ligand**<br>**Water**	 78.95<br>81.23<br>44.30
**Model composition**<br>**Non-hydrogen atoms**<br>**Protein residues**<br>**Ligands**<br>**Water**	 7237<br>880<br>42<br>412
**R.m.s. deviations**<br>**Bond lengths (Å)**<br>**Bond angles (°)**	 0.02<br>1.59
**Ramachandran plot**<br>**Favored (%)**<br>**Allowed (%)**<br>**Disallowed (%)**	 89.36<br>9.04<br>1.60
**Validation**<br>**Clashscore**	 17.05

bioRxiv preprint doi: https://doi.org/10.64898/2026.09.07.749649; this version posted September 10, 2026. The copyright holder for this preprint (which was not certified by peer review) is the author/funder, who has granted bioRxiv a license to display the preprint in perpetuity. It is made available under a CC-BY 4.0 International license.

**Poor rotamers (%)**	1.83

Table S3. X-ray crystallography data collection, processing and model building statistics for the PVA269 Fab-bound HPIV3 HN head structure.

**Ligand**	**Analyte**	**K_D (nM)**	**k_{on} (1/Ms)**	**k_{off} (1/s)**
HPIV2 HN WT	IgG	0.017<br>0.015	5.47E+05<br>7.05E+05	9.34E-06<br>1.07E-05
	Fab	15.35<br>17.42	5.41E+05<br>4.60E+05	6.93E-03<br>8.00E-03
HPIV2 HN R354A	IgG	0.686<br>0.647	5.23E+05<br>5.53E+05	3.59E-04<br>3.58E-04
	Fab	ND	ND	ND

Table S4. BLI K_D, on rate (K_{on}), and off rates (K_{off}) of two biological replicates of HPIV2 HN wildtype (WT) or two technical replicates of R354A mutant with PVA269 IgG or Fab. ND = not determined due to very weak binding.
